# Histone Deacetylase Inhibitor Phenylbutyrate Exaggerates Heart Failure in Pressure Overloaded Mice independently of HDAC inhibition

**DOI:** 10.1038/srep34036

**Published:** 2016-09-26

**Authors:** Jing Ma, Tao Luo, Zhi Zeng, Haiying Fu, Yoshihiro Asano, Yulin Liao, Tetsuo Minamino, Masafumi Kitakaze

**Affiliations:** 1 Department of Cardiology, Chinese PLA General Hospital, Beijing, China; 2 Department of Cardiovascular Medicine, Osaka University Graduate School of Medicine, Suita, Osaka, Japan; 3 Department of Cardiology, Nanfang Hospital, Southern Medical University, Guangzhou, China; 4 Cardiovascular Division of the Department of Medicine, National Cerebral and Cardiovascular Center, Osaka, Japan

## Abstract

4-Sodium phenylbutyrate (PBA) has been reported to inhibit endoplasmic reticulum stress and histone deacetylation (HDAC), both of which are novel therapeutic targets for cardiac hypertrophy and heart failure. However, it is unclear whether PBA can improve heart function. Here, we tested the effects of PBA and some other HDAC inhibitors on cardiac dysfunction induced by pressure overload. Transverse aortic constriction (TAC) was performed on male C57BL/6 mice. PBA treatment (100 mg/kg, 6 weeks) unexpectedly led to a higher mortality, exacerbated cardiac remodelling and dysfunction. Similar results were noted in TAC mice treated with butyrate sodium (BS), a PBA analogue. In contrast, other HDAC inhibitors, valproic acid (VAL) and trichostatin A (TSA), improved the survival. All four HDAC inhibitors induced histone H3 acetylation and inhibited HDAC total activity. An individual HDAC activity assay showed that rather than class II_a_ members (HDAC4 and 7), PBA and BS predominantly inhibited class I members (HDAC2 and 8), whereas VAL and TSA inhibited all of them. These findings indicate that PBA and BS accelerate cardiac hypertrophy and dysfunction, whereas VAL and TSA have opposing effects.

Maladaptive hypertrophy often leads to cardiac dilation and heart failure (HF)^1^. HF is becoming the predominant cardiovascular disorder worldwide, provoking a therapeutic challenge because of the suboptimal efficacy of existing therapies. Recent studies by us and others have revealed that endoplasmic reticulum (ER) stress and histone acetylation may work as a nodal control point in the complex signalling network for the regulation of cardiac hypertrophy and HF^2^,^3^,^4^,^5^. 4-Phenylbutyrate acid (PBA), a short-chain fatty acid, which is currently in clinical application in patients with hematologic malignancies or refractory solid tumours^6^,^7^,^8^, has been reported to inhibit ER stress and histone acetylation^9^,^10^,^11^, but it is largely unknown whether PBA exerts any effects on cardiac hypertrophy and HF.

In addition to their anti-tumour effects, histone deacetylation (HDAC) inhibitors have recently caught the attention of cardiologists because some inhibitors such as trichostatin A (TSA), valproic acid (VAL), SK-7014 and scriptaid have been reported to blunt pressure overload-induced cardiac hypertrophy in mice^12^,^13^, which may provide a novel therapeutic strategy for HF. Based on the fact that clinical application of many anti-neoplastic agents has been hampered by their toxicity^14^, it would be of great prospect if PBA could also exhibit beneficial effects in patients with heart diseases, because it is increasingly probable that a patient may coincidently suffer from both cancer and cardiovascular disease.

HDACs are grouped into four classes: class I (HDAC1, 2, 3 and 8), II_a_ (HDAC4, 5, 7 and 9) and II_b_ (HDAC6 and 10), class III (sirtuin enzymes 1–7), and class IV (HDAC11–15). Their roles in the pathophysiology of cardiac hypertrophy and failure are controversial or unknown. It is generally believed that activation of class I members is pro-hypertrophic, while activation of class II_a_ suppresses cardiac hypertrophy^12^,^15^,^16^,^17^,^18^, implicating that agents capable of inhibiting class I and activating class II_a_ HDACs would be optimal for anti-hypertrophic therapy. Paradoxically, class I and II broad-spectrum inhibitors have been demonstrated to blunt cardiac hypertrophy^12^,^13^, suggesting that class II_a_ HDACs are relatively insensitive to commonly used HDAC inhibitors, and class I HDACs play a dominant role in governing the hypertrophic process.

In this study, we noted that PBA and its analogue, butyrate sodium (BS), exert strong inhibitory effects on class I and weak suppression of class II_a_ HDACs in cardiomyocytes. Therefore, we hypothesized that PBA may be optimal for blunting cardiac hypertrophy and the subsequent HF induced by transverse aortic constriction (TAC) operation. Surprisingly, our study demonstrated that PBA deteriorated cardiac remodelling and dysfunction. Similarly, BS increased the mortality of mice after TAC operation, which was contrary to the data from mice treated with TSA or VAL. Unlike the beneficial effects of other class I and II broad-spectrum inhibitors, our findings indicated for the first time that PBA is detrimental to hypertrophic and failing heart induced by pressure overload, suggesting that caution should be given to its clinical application in related cardiovascular disease.

## Results

### HDAC inhibitors have different selectivity to class I and II_a_ HDACs

**Figure 1:**
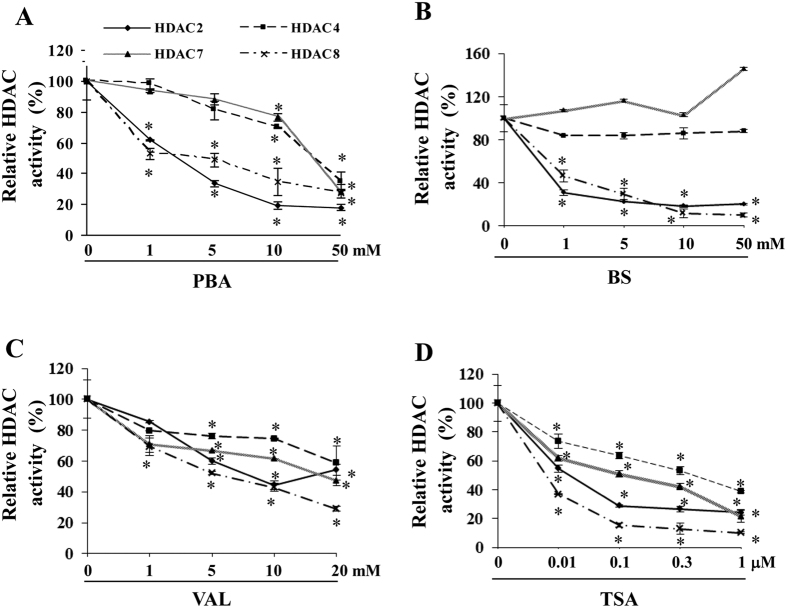
Inhibition of different HDAC subtypes by individual inhibitors in neonatal rat cardiomyocytes. (**A–D**) Active recombinant HDAC2, 4, 7 or 8 was incubated with a specific unique fluorogenic substrate in the presence or absence of the HDAC inhibitor, PBA (**A**), BS (**B**), TSA (**C**) or VAL (**D**), at different doses. The system’s fluorescence intensity was measured using a fluorescence reader after adding Lysine Developer. Data are expressed as mean ± SEM; n = 3; *P < 0.05 vs. the corresponding baseline of each HDAC subtype.

We investigated the inhibitory spectrum of PBA, BS, VAL and TSA with regard to individual HDAC2, 4, 7 and 8. All four inhibitors inhibited HDAC2 and 8 (class I) potently, while they differed a lot in regard to HDAC4 and 7 (class II_a_) (Fig. 1). Similar to their effects on class I HDACs, VAL and TSA also robustly inhibited HDAC4 and 7 (Fig. 1C,D). In contrast, PBA and BS had modest or even no obvious inhibitory effect on these class II_a_ HDACs (Fig. 1A,B), although toxic doses of PBA (>25 mM) significantly inhibited them. Because it has been proposed that activation of class I members is pro-hypertrophic, while activation of class II_a_ suppresses cardiac hypertrophy^12^,^15^,^16^,^17^,^18^, we postulated that PBA should have a more potent anti-hypertrophic effect than nonselective HDAC inhibitors. Thus, we next determined the role of PBA in pressure overload-induced cardiac remodelling.

### PBA unexpectedly enhanced cardiac hypertrophy

**Figure 2:**
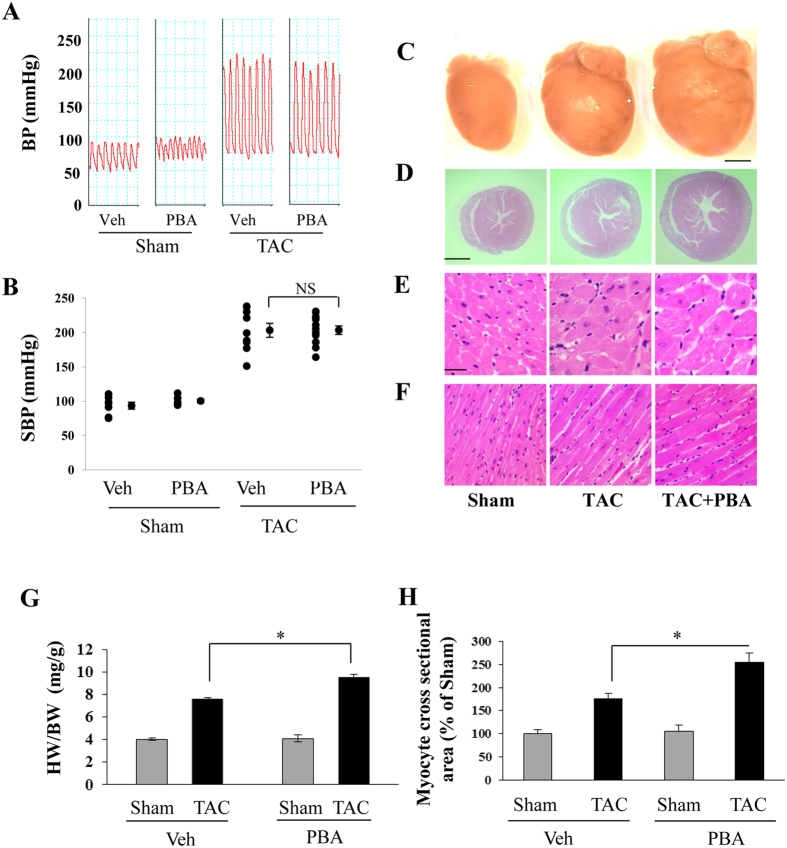
Effects of PBA on cardiac hypertrophy induced by pressure overload in mice. (**A**) Representative recording curves of blood pressure (BP). (**B**) Statistical results of systolic BP (SBP) recorded from the right carotid artery by invasive cannulation with a Millar Pressure Catheter in mice subjected to sham or TAC operation with or without PBA treatment (100 mg/kg/d, ip, for 6 weeks). Sham + Veh (n = 7); Sham + PBA (100 mg/kg/d, ip, n = 6); TAC (n = 9); TAC + PBA (100 mg/kg/d, ip, n = 12). (**C**) Representative images of whole hearts. (**D**) Whole view of heart cross sections stained with haematoxylin–eosin. (**E**) Cross-axis view of cardiomyocytes. (**F**) Long-axis view of cardiomyocytes. Scale = 20 μm in **D** and **E**. (**G**) The ratio of heart weight (HW) to body weight (BW). (**H**) Cardiomyocyte cross-sectional area. In (**G**) TAC group (n = 22), TAC + PBA group (n = 22), Sham group (n = 8), Sham + PBA group (n = 8). In (**H**) four mice in each group were selected and 400 cardiomyocytes per animal were chosen randomly. Data are expressed as mean ± SEM. *P < 0.05. NS, not significant; TAC, transverse aortic constriction; Veh, vehicle.

The systolic blood pressure of the aorta in both the TAC and TAC + PBA (100 mg/kg/d) groups reached about 200 mmHg (Fig. 2A,B), indicating that similar pressure overload between the two groups was achieved. Six weeks later, TAC resulted in a significant increase in whole heart size (Fig. 2C), left ventricular (LV) wall thickness (Fig. 2D), cross section area of LV cardiomyocytes (Fig. 2E) and muscle bundle size of LV myocardium (Fig. 2F). Compared with the Sham group, a 90% increase in the heart weight/body weight (HW/BW) ratio (Fig. 2G) and a marked enlarged cross-sectional surface area of cardiomyocytes were noted in the TAC group (Fig. 2H). Unexpectedly, PBA treatment further amplified the cardiac hypertrophy (Fig. 2C–H). The HW/BW ratio increased to 138% of the sham mice (Fig. 2G), which was also evidenced by histological findings (Fig. 2H). There were no significant differences in the HW/BW ratio and the cardiomyocyte cross-sectional surface area between PBA-treated and untreated sham mice.

### Larger cardiac remodelling and lower systolic function in PBA-treated TAC mice

**Figure 3:**
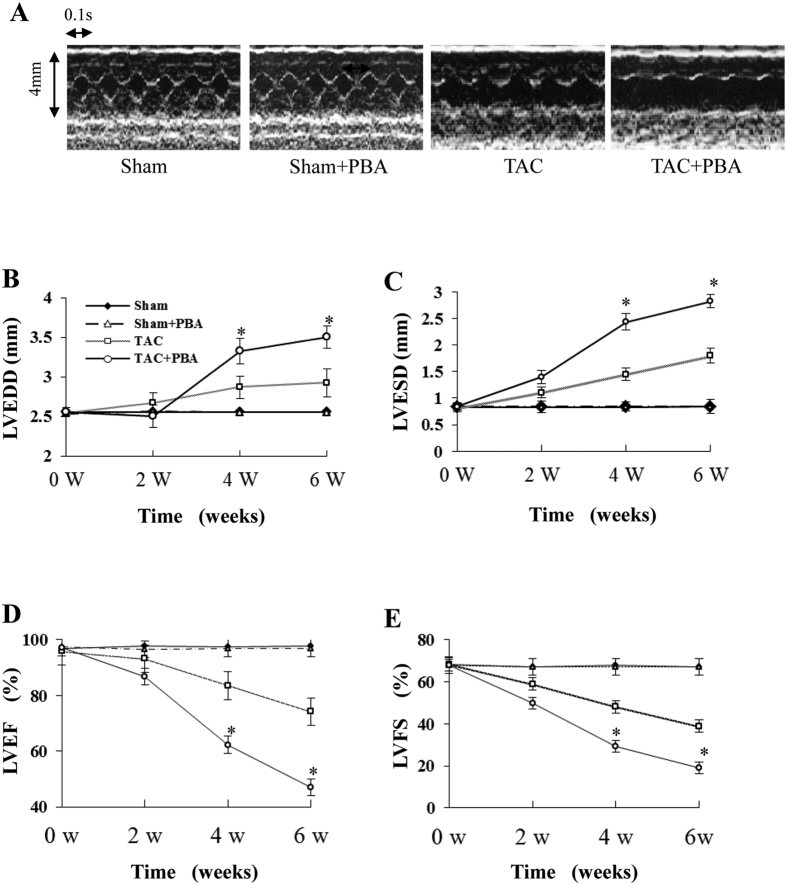
Time course results of echocardiographic examination of pressure-overloaded mice treated with PBA. (**A**) Representative pictures of M-mode echocardiography. (**B**) Left ventricular end-diastolic diameter (LVEDD). (**C**) Left ventricular end-systolic diameter (LVESD). (**D**) Left ventricular ejection fraction (LVEF). (**E**) Left ventricular fractional shortening (LVFS). Sham (n = 10), Sham + PBA (n = 10), TAC (n = 14 at 2 w, n = 10 at 4 w and n = 7 at 6 w) and TAC + PBA (n = 20 at 2 w, n = 12 at 4 w and n = 6 at 6 w). Data are expressed as mean ± SEM. *P < 0.05 vs. the values of the same time point of the TAC group. TAC, transverse aortic constriction.

In a time course experiment, we noted a marked LV chamber dilation (Fig. 3A–C), and a significant decrease in both LV fractional shortening (LVFS) and the LV ejection fraction (LVEF) over time in the PBA (100 mg/kg/d)-treated TAC mice (Fig. 3D,E) compared with the untreated TAC mice. In contrast, there were no significant differences in these parameters between PBA-treated and untreated sham mice. These findings indicate that the increase in cardiac remodelling under pressure overload was promoted by PBA.

### PBA impairs left ventricular haemodynamics

**Figure 4:**
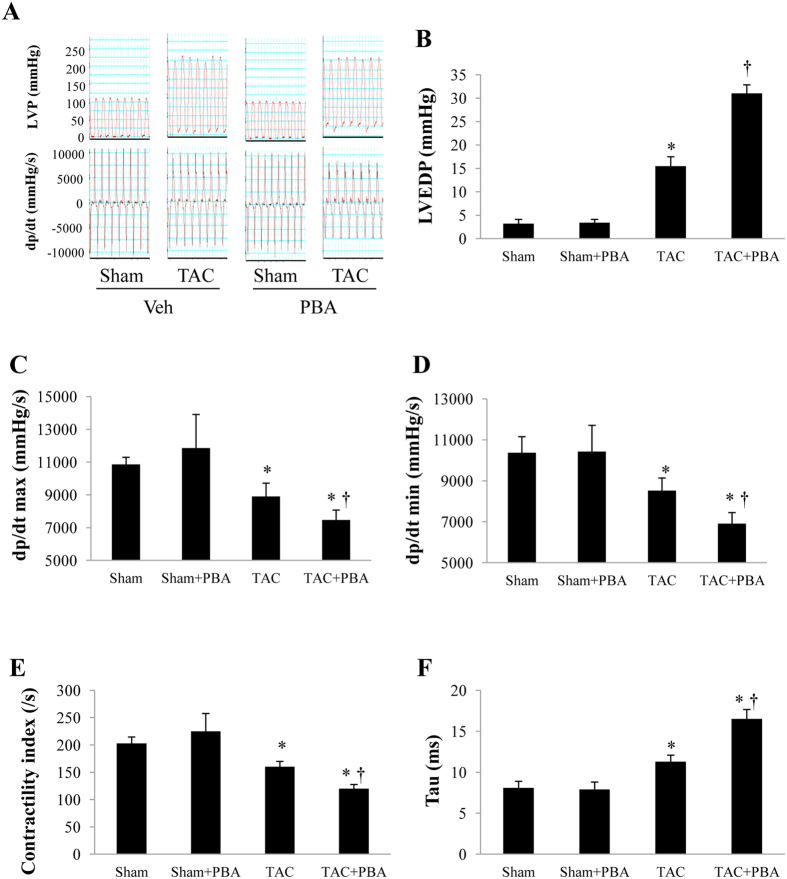
Results of left ventricular haemodynamics 6 weeks after surgery. (**A**) Representative recording curves of left ventricular pressure (LVP). (**B**) Left ventricular end-diastolic pressure (LVEDP). (**C**) The maximum rising rate of LVP (d*P*/d*t* max). (**D**) The maximum fall rate of LVP (d*P*/d*t* min). (**E**) LV contractility index. (**F**) The exponential time constant of relaxation (tau). Data are expressed as mean ± SEM. *P < 0.05 vs. the Sham group, ^†^*P* < 0.05 vs. the TAC group. Sham + Veh (n = 7); Sham + PBA (100 mg/kg/d, ip, n = 6); TAC (n = 9); TAC + PBA (100 mg/kg/d, ip, n = 12). TAC, transverse aortic constriction; Veh, vehicle.

Six weeks after surgery, the LV systolic pressure was similar in PBA-treated and untreated TAC mice. However, both the LV end-diastolic pressure (LVEDP) and Tau were significantly larger, and the ± d*P*/d*t* max and LV contractility index were significantly lower in PBA (100 mg/kg/d)-treated TAC mice than in the vehicle-treated group (Fig. 4A–F, Table 1). No significant change was found in the PBA-treated sham mice (Table 1). These results suggest that PBA worsened both the systolic and diastolic function in TAC mice.

### PBA increases mortality and pulmonary congestion

**Figure 5:**
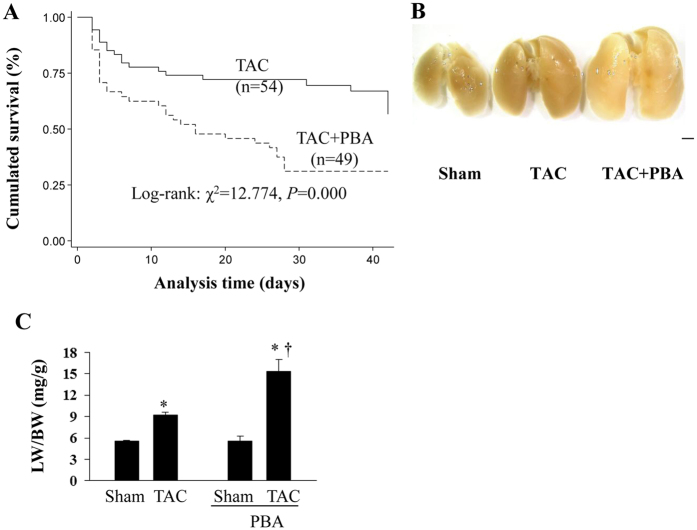
PBA increases mortality and pulmonary congestion in mice with TAC surgery. (**A**) Kaplan–Meier analysis of the mortality rate of TAC mice treated with or without PBA (100 mg/kg/d, ip, for 6 weeks). (**B**) Representative pictures of the lungs. (**C**) Lung weight (LW) to body weight (BW). Scale bar: 2 mm. Data are expressed as mean ± SEM. *P < 0.05 vs. the Sham group, ^†^*P* < 0.05 vs. the TAC group. TAC group (n = 22), TAC + PBA group (n = 22), Sham group (n = 8), Sham + PBA group (n = 8). TAC, transverse aortic constriction.

In response to high-pressure overload, some mice died of severe acute or chronic heart failure. Lung haemorrhage or pleural effusion was also seen in some cases. Kaplan–Meier analysis revealed that the survival rate of vehicle-treated TAC mice was 66.7% at 6 weeks after surgery, while it was only 32.7% in the PBA-treated (100 mg/kg/d) group (*P* < 0.01, Fig. 5A).

Compared with the sham mice, the lung-to-body weight ratio (LW/BW) increased by 170.8% in the PBA-treated TAC mice, whereas there was only a 50.3% increase in the vehicle-treated TAC group (Fig. 5B,C). There was no significant difference in the LW/BW between the PBA- and vehicle-treated sham mice.

Collectively, these results demonstrated that PBA worsened cardiac hypertrophy and failure in the context of pressure overload, while in the sham mice PBA did not exhibit any effect on cardiac function, suggesting that the unfavourable effect of PBA may be specific to the pathological process. We next examined the effects of PBA on several hypertrophy-related genes and proteins.

### PBA affects ANP, BNP, histone H3 acetylation, CHOP and JNK activity

**Figure 6:**
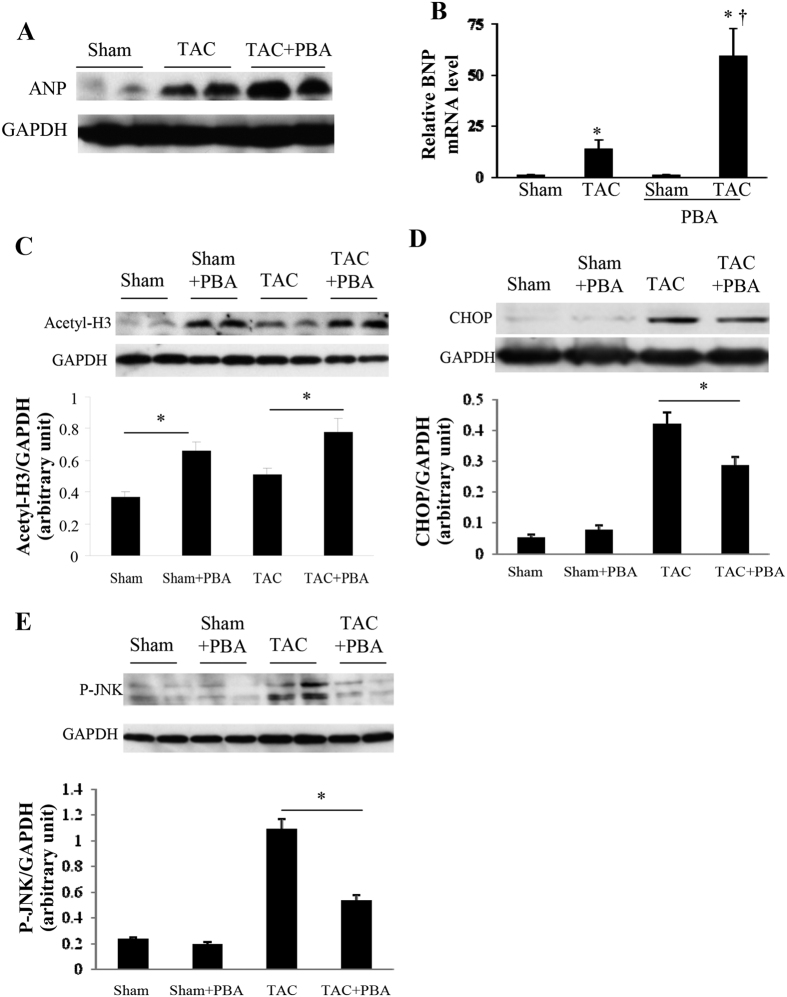
Effects of PBA on the expression of ANP, BNP, histone H3 acetylation, CHOP and JNK activity 6 weeks after surgery. (**A**) Representative western blots of myocardial ANP (atrial natriuretic peptide; cropped from Fig. S1A). n = 4 in each group. (**B**) mRNA level of *BNP* (brain natriuretic peptide) determined by real-time PCR. n = 4 in each group. *P < 0.05 vs. the Sham group, ^†^*P* < 0.05 vs. the TAC group. (**C–E**) Western blots of myocardial histone H3 (H3) acetylation (**C**) C/EBP homologous protein (CHOP) (**D**) and JNK phosphorylation (**E**). These blots were cropped from Fig. S1B–D, respectively. n = 4 in each group. *P < 0.05. Data are expressed as mean ± SEM. TAC, transverse aortic constriction. All the gels have been run under the same experimental conditions.

TAC surgery remarkably increased the cardiac expression levels of the hypertrophic markers, atrial natriuretic factor (ANP) and brain natriuretic peptide (BNP), while PBA (100 mg/kg/d) remarkably augmented these upregulation effects (Fig. 6A,B). The acetylated histone H3 levels were significantly elevated in PBA-treated TAC or sham mice (Fig. 6C), indicating that PBA successfully inhibited the total HDAC activity. TAC mice also displayed an increased cardiac acetylated histone H3 (H3) level compared with the sham mice (Fig. 6C). C/EBP homologous protein (CHOP), an ER stress-related protein, was significantly induced in the TAC group and was moderately but significantly inhibited by PBA treatment (Fig. 6D). The phosphorylation of c-Jun N-terminal kinase (JNK), a recently reported anti-hypertrophic factor^19^, was markedly increased in TAC mice and was robustly downregulated by PBA treatment (Fig. 6E).

To exclude the possibility that a non-specific function of PBA contributes to its detrimental effects on pressure-overloaded heart, we used BS, an analogue of PBA, and the broad spectrum HDAC inhibitors, TSA and VAL, to comparably examine their effects in TAC mice.

### Distinct effects of HDAC inhibitors on pressure-overloaded heart

**Figure 7:**
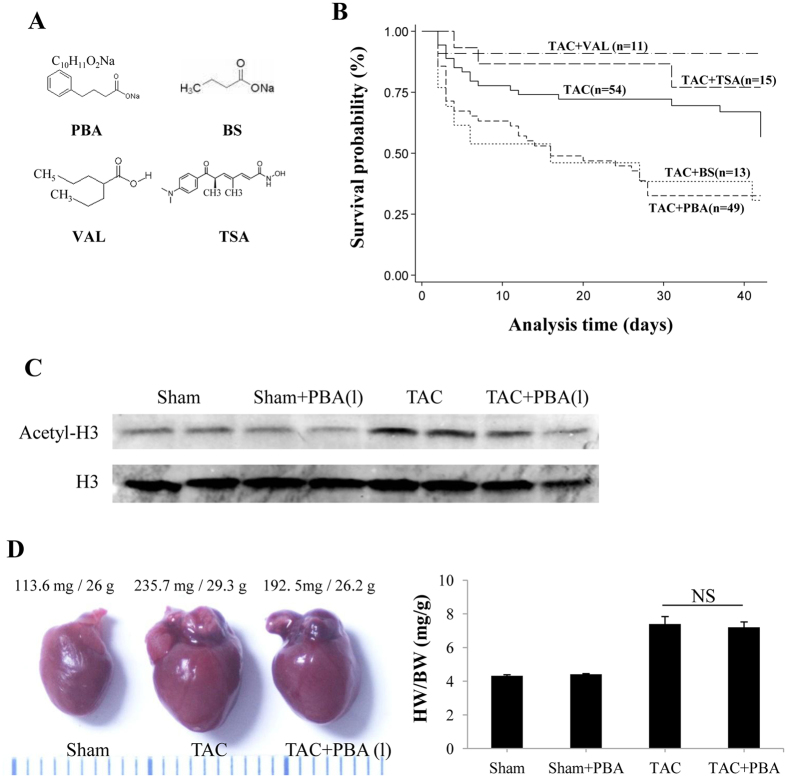
The effects of different HDAC inhibitors on TAC-induced mortality. (**A**) The chemical structures of the HDAC inhibitors, PBA, BS, TSA and VAL. (**B**) Kaplan–Meier analysis of the mortality rate of mice subjected to TAC surgery with or without HDAC inhibitor treatment for 6 weeks, including PBA (100 mg/kg/d, ip), BS (100 mg/kg/d, ip), TSA (0.6 mg/kg/d, ip) and VAL (0.71% in drinking water). (**C**) Western blots of H3 acetylation. (**D**) Representative image of whole hearts (left panel), and ratio of heart weight (HW) to body weight (BW) (right panel) of the TAC mice with or without low-dose PBA treatment (10 mg/kg/d, ip, for 6 weeks). Data are expressed as mean ± SEM. Sham + Veh (n = 4); Sham + PBA (10 mg/kg/d, ip, n = 4); TAC (n = 6); TAC + PBA (10 mg/kg/d, ip, n = 6). PBA (1), PBA 10 mg/kg/d; TAC, transverse aortic constriction.

The chemical structure of PBA, BS, TSA and VAL is shown in Fig. 7A. Interestingly, BS also significantly increased the mortality of TAC mice. The survival rate was similar to that of PBA-treated TAC mice (30.8% vs. 32.7%, Fig. 7B). In contrast, either VAL or TSA treatment significantly improved the survival rate of TAC mice, which is in good agreement with previous reports^12^,^13^.

We noted that a low dose of PBA (10 mg/kg/d) did not induce H3 acetylation (Fig. 7C), and exerted no significant influence on cardiac hypertrophy and heart failure in pressure-overloaded mice (Fig. 7D).

### Similar effect of different HDAC inhibitors on H3 acetylation in cardiomyocytes

**Figure 8:**
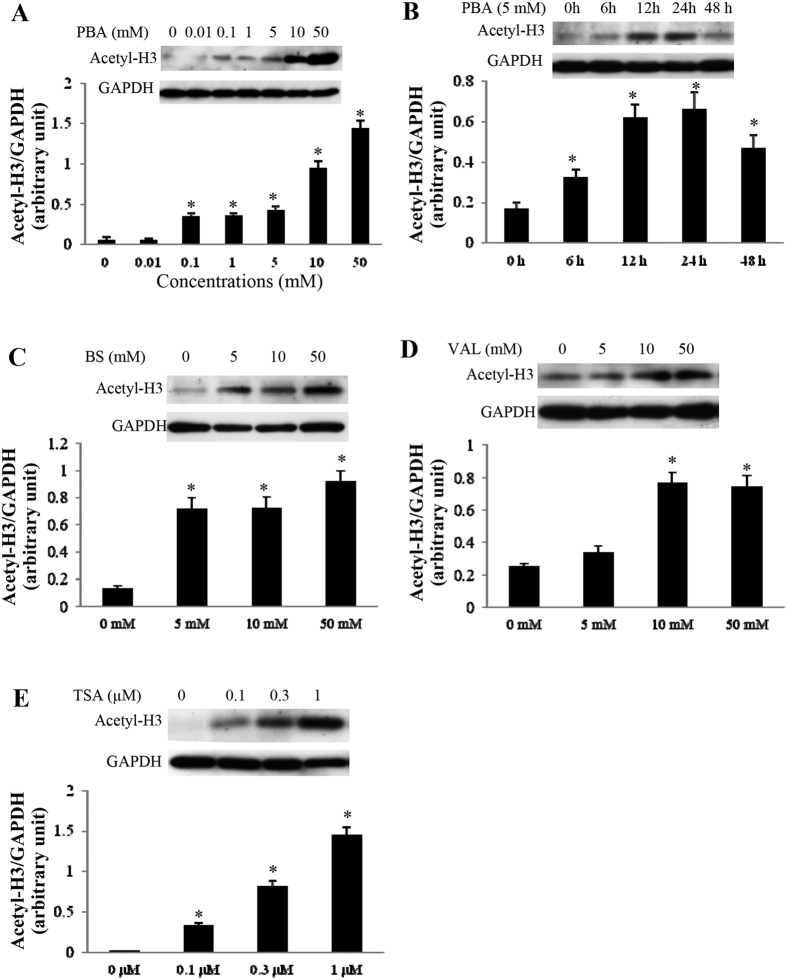
The effects of different HDAC inhibitors on H3 Acetylation. (**A**,**B**) Western blots of H3 acetylation in cultured neonatal cardiomyocytes treated with different concentrations of PBA for 24 h (**A**) or with 5 mM PBA for different periods (0, 6, 12, 24 and 48 h) (**B**). n = 4 in each group. (**C–E**) Western blots of H3 acetylation in cultured neonatal cardiomyocytes treated with different concentrations of BS (**C**), VAL (**D**) or TSA (**E**) for 24 h. n = 4 in each group. Data are expressed as mean ± SEM; *P < 0.05 vs. baseline. The blots in (**A**–**E**) were cropped from Fig. S1E–I, respectively. All the gels were run under the same experimental conditions.

The acetylated H3 levels in the cultured cardiomyocytes were analysed by western blotting. As shown in Fig. 8A,B, PBA treatment inhibited HDAC activity in a time- and dose-dependent manner. Exposure to PBA, even at a low dose of 0.1 mmol/L, induced hyperacetylation of H3. Similarly, BS, VAL and TSA promoted H3 acetylation in a dose-dependent manner (Fig. 8C–E).

## Discussion

HDACs have drawn the attention of cardiovascular researchers, because of their potential role in controlling hypertrophic gene expression in the myocardium. To date, several lines of evidence have demonstrated that HDACs in cardiomyocytes indeed play critical roles in the heart, and HDAC inhibitors seem to hold promise in the treatment of pressure overload-induced cardiac hypertrophy^12^,^15^,^16^,^17^,^18^. In this study, we demonstrated, for the first time, that the HDAC inhibitor, PBA, and its analogue, BS, promoted cardiac hypertrophy and exacerbated heart failure in response to pressure overload, which was opposite to the pan-HDAC inhibitors, TSA and VAL. In addition, the survival rate significantly decreased by treatment with PBA or BS, and improved by TSA or VAL. These findings challenge the notion that activation of class I and class II_a_ HDACs is pro-hypertrophic and anti-hypertrophic, respectively^12^,^15^,^16^,^17^,^18^,^20^,^21^, because both PBA and BS have higher selectivity to inhibit class I HDACs, and thus should exert a more potent anti-hypertrophic effect.

PBA and BS are far less potent than other HDAC inhibitors, as only at millimolar concentrations do they inhibit HDACs *in vivo* by a noncompetitive mechanism^22^,^23^, therefore we used a high dose of these two agents (100 mg/kg/d, while TSA was 0.6 mg/kg/d). In this study, despite the fact that PBA, BS, TSA and VAL increased H3 acetylation levels, their effects on cardiac remodelling and failure were totally distinct. It may be partially ascribed to their different effect on certain HDAC members^20^. Although VAL has been reported to be a class I HDAC-selective inhibitor in Hela cells^24^, substantial evidence supports the idea that VAL also inhibits class II HDAC members such as HDAC4, 5, 7 and 6 in other types of cells and tissues^25^,^26^,^27^,^28^,^29^. The present study suggests that VAL is not a specific class I HDAC inhibitor in cardiomyocytes, but it exhibits features of a pan-HDAC inhibitor similar to TSA. Similarly, Kee *et al*. have observed that VAL blunted cardiac hypertrophy by inhibiting the activity of class II_b_ HDAC6 and class I HDAC8^29^. In addition, different regulation of gene and protein function may be a potential mechanism. Substantial evidence has revealed that HDACs work as a stress-signal responsive element to regulate gene expression, both positively and negatively^17^,^18^,^30^,^31^. HDAC inhibitors heterogeneously alter the transcription of a large set of genes that control diverse molecular pathways important for cell survival and proliferation. Moreover, many hypertrophy-related non-histone proteins are regulated by HDAC inhibitors^32^, including GATA4^33^, serum response factor (SRF)^34^, nuclear factor of activated T cells (NFAT)^35^, NK family transcription factors^36^ and myocyte enhancer factor-2 (MEF2)^37^. It is quite likely that the regulation of pressure overload-induced cardiac hypertrophy by HDAC molecules is much more complex than we expected. Because of the different chemical conformation and catalytic position, HDAC inhibitors have been shown to hold a distinct inhibitory spectrum. In this study, we noted that rather than class II_a_ members (HDAC4 and 7), PBA and BS predominantly inhibited class I members (HDAC2 and 8), whereas VAL and TSA inhibited all of them to a similar extent. These results imply that the detrimental effects of PBA and BS were caused by other nonspecific actions irrelevant to HDAC inhibition. PBA has been reported to increase the production of the proinflammatory cytokine, interleukin-8, in lung epithelial cells and to inhibit angiogenesis in prostate tumours^38^,^39^, both of which can promote the progression of cardiac hypertrophy and heart failure in the context of cardiovascular pathophysiology. New evidence has shown that PBA antagonized doxorubicin-induced cardiomyocyte death and cardiac atrophy^40^, supporting the idea that PBA promotes cardiomyocyte growth.

The precise role of individual HDACs in regulating cardiac growth gene expression remains largely unknown, despite some clues from gene-manipulation studies. We cannot exclude the possibility of compensatory activation of redundant HDACs when one HDAC subtype is deleted, because studies with knockout mice may reflect the role of the presence of the enzymes rather than their enzymatic activity.

PBA has been reported to inhibit ER stress in cardiomyocytes and noncardiomyocytes^9^,^10^. Here, we confirmed that PBA moderately reduced CHOP expression. Although ER stress contributes to the progression of cardiac remodelling^3^,^4^,^5^, in the context of pressure overload, the inhibition on ER stress by PBA is not sufficient to balance out its detrimental effects. The worsening of heart failure by PBA may also be ascribed to its inhibitory effect on JNK phosphorylation, as new evidence suggests that JNK activation reduces hypertrophy and prevents transition to heart failure by antagonizing calcineurin-NFAT dephosphorylation^19^. It is generally accepted that activation of the JNK pathway is mainly anti-hypertrophic^41^,^42^.

The deterioration of heart function by PBA administration may also be partially ascribed to reactivation of fetal gene programs, such as ANP and BNP. Furthermore, PBA did not affect the blood pressure of TAC or sham-operated mice, excluding the possibility that its detrimental role is secondary to the increase in afterload. Considering the various cellular and molecular mechanisms of PBA may confound the interpretation of the present study, we investigated the effect of its analogue, BS, a recognized classical HDAC inhibitor, and obtained similar results. Moreover, the dose of PBA and BS used in this study was tolerated well by sham mice, ruling out toxicity.

The anti-hypertrophic mechanisms of VAL and TSA have been demonstrated in previous studies. VAL has been reported to blunt cardiac hypertrophy by inhibiting the activity of HDAC6^29^ or by both inhibiting HDAC catalytic activity and inducing specific degradation of HDAC2^22^. Majumdar *et al*. have reported that TSA caused significant induction of phosphatase and tensin homolog expression to counteract interleukin-18-induced proinflammatory signalling and cardiac hypertrophy^43^, while Kee *et al*. have reported that TSA-induced prevention of cardiac hypertrophy was mediated by Krüppel-like factor 4, a novel anti-hypertrophic transcriptional regulator^44^.

In summary, we propose that the HDAC inhibitors at the doses we employed in this study, PBA and BS, enhance pressure overload-induced cardiac dysfunction and increase mortality, whereas VAL and TSA have opposing effects. The dual regulatory effect of HDAC inhibitors on pressure overload-induced cardiac dysfunction improves our comprehension about the complex effect of HDAC inhibitors and warns us that only some HDAC inhibitors are beneficial for pressure overload-induced cardiac dysfunction. The worsening of cardiac remodelling by PBA at a high dose is likely mediated through an “off target” effect such as JNK inactivation, rather than through HDAC inhibition.

## Methods

All procedures involving experimental animals were performed in accordance with the protocols approved by the Committee for Animal Research of Southern Medical University and the Committee for Animal Research of Osaka University, and conformed to the “Position of the American Heart Association on Research Animal Use” adopted by the AHA on November 11, 1984.

### Cell culture

Neonatal rat (aged 2–3 days) ventricular myocytes were isolated and cultured as we described previously^45^,^46^. Cells were exposed to PBA, BS, VAL or TSA at the indicated concentration for 24 h, except for the cardiomyocytes used for the PBA time course experiment, which were treated for the indicated time.

### TAC model and experimental protocols

Male C57BL/6 mice (7–8 weeks old, weighing 18–23 g) were anesthetized with an intraperitoneal (ip) injection of 50 mg/kg pentobarbital and TAC preparation was performed as described elsewhere^47^,^48^.

TAC mice were randomly divided into five groups: (1) TAC group (n = 54), saline was used as vehicle; (2) TAC + PBA group (n = 49), PBA 100 mg/kg/d dissolved in saline, ip; (3) TAC + BS group (n = 13), BS 100 mg/kg/d, ip; (4) TAC + TSA group (n = 15), TSA 0.6 mg/kg/d daily, ip; (5) TAC + VAL group (n = 9), VAL at a dose of 0.71% in drinking water. The dose and administration method of TSA and VAL were according to a previous study^12^. Accordingly, the mice receiving sham surgery were also randomly divided into Sham, Sham + PBA, Sham + BS, Sham + TSA and Sham + VAL groups, n = 8 in each group. The dose of PBA was decided according to a previous study^40^,^49^. A low dose of PBA (10 mg/kg/d) was also used to investigate its effect on cardiac hypertrophy.

### Echocardiography

To exclude the influence imposed by the anaesthesia drug, mice underwent transthoracic echocardiography in a conscious state with a Sonos 4500 and a 15–6 L MHz transducer (Philips, Amsterdam, the Netherlands) over time as described elsewhere^48^,^50^.

### Histological analysis

Mice were euthanized 6 weeks after TAC, and the hearts were rinsed with saline and placed in 10% formalin. The cell surface area was quantified using four hearts from each group, as described previously^4^,^51^.

### Invasive measurement of haemodynamics

At week 6 after aortic banding, mice were lightly anesthetized with phenobarbital (30 mg/kg) and underwent endotracheal intubation. A 1.4 F Millar pressure catheter (Millar Instruments) was inserted into the right carotid artery, and further advanced into the left ventricle. The carotid artery pressure and left ventricular pressure were measured simultaneously and subsequently analysed with a data acquisition and analysis system (PowerLab, AD Instruments).

### RNA preparation and analysis

Total RNA of homogenized mouse whole heart was prepared using RNA-Bee isolation reagent (Tel-Test, Inc.) according to the protocol of the manufacturer. Reverse transcription PCR (RT-PCR) was performed. *ANP* and *BNP* expression was determined using real-time PCR with an ABI PRISM 7000 Sequence Detection System (Applied Biosystems). Glyceraldehyde-3- phosphate dehydrogenase (*GAPDH*) was used as internal control.

### Western blotting

Total proteins were prepared from whole heart tissue homogenate or cultured cardiomyocytes. Immunoblotting was performed using antibodies directed against acetylated histone H3 (Upstate) and GAPDH. Immunoreactive bands were visualized by enhanced chemiluminescence (Amersham) and quantified by densitometry with Scion Image software.

### HDAC activity assay

Enzyme-active recombinant HDAC4, 7 and 8 were purchased from BPS Bioscience Inc., and HDAC2 was purchased from BIOMOL. The HDAC4 and HDAC7 assays were performed by Fluorogenic HDAC Class 2a Assay Kit (BPS Bioscience), and the HDAC2 and HDAC8 assays were performed by Fluorogenic HDAC Assay Kit (BPS Bioscience). The specific HDAC fluorometric substrate, comprised of an acetylated lysine side chain, was incubated with purified individual HDAC enzymes (HDAC2, 4, 7 and 8), in the presence or absence of the HDAC inhibitors, PBA, BS, TSA or VAL, at different doses. After adding Lysine Developer, the fluorophore that could then be measured using the fluorescence plate reader Spectra (Max Gemini, Molecular Devices, Sunnyvale, CA), was excitated at a wave-length of 365 nm and detected by emitted light at 450 nm.

### Statistical analysis

Data are reported as mean ± SEM. Statistical significance was analysed using a Student unpaired *t* test or one-way ANOVA followed by the Bonferroni method for *post hoc* pairwise multiple comparisons. Survival analysis was performed using the Kaplan–Meier method. *P* < 0.05 was considered to indicate statistical significance.

The authors had full access to the data and take full responsibility for its integrity. All authors have read and agree to the manuscript as written.

## Additional Information

**How to cite this article**: Ma, J. *et al*. Histone Deacetylase Inhibitor Phenylbutyrate Exaggerates Heart Failure in Pressure Overloaded Mice independently of HDAC inhibition. *Sci. Rep.* **6**, 34036; doi: 10.1038/srep34036 (2016).

## Supplementary Material

Supplementary Information

## Figures and Tables

**Table 1:** LV Hemodynamics at 6 weeks after TAC or Sham Surgery.

	Sham	Sham + PBA	TAC	TAC + PBA
LVESP, mmHg	105 ± 3.5	105 ± 3.9	203 ± 11.2†	203 ± 9.3†
LVEDP, mmHg	3.2 ± 0.91	3.4 ± 0.71	15.5 ± 2.00†	31.0 ± 1.85†,#
Contractivity index, 1/s	203 ± 11.6	225 ± 32.6	160 ± 9.9*	120 ± 7.5*,§
τ, ms	8.1 ± 0.80	7.9 ± 0.90	11.3 ± 0.79*	16.5 ± 1.16*,§
Max d*P*/d*t*, mmHg/s	10861 ± 433.2	11853 ± 2051.5	8900 ± 814.7	7472 ± 602.9
Min d*P*/d*t*, mmHg/s	−10367 ± 783.4	−10433 ± 1275.7	−8519 ± 619.3	−6913 ± 537.3
HR, bpm	509 ± 25	542 ± 29	422 ± 22	427 ± 17

TAC, transverse aortic constriction; HR, heart rate; LVESP, maximum left ventricular end-systolic pressure; LVEDP, left ventricular end-diastolic pressure; Contractility index: Max dP/dt divided by the pressure at the time of Max dP/dt; Tau, the exponential time constant of relaxation; Max dP/dt, the steepest slope during the upstroke of the pressure curve; Min dP/dt, the steepest slope during the downstroke of the pressure curve; Sham (n = 6), Sham + PBA (100 mg/kg/d, n = 6), TAC (n = 10) and TAC + PBA (100 mg/kg/d, n = 8). Data are mean ± S.E.M. ^*^P < 0.05, ^**†**^*P* < 0.001 vs. Sham. ^§^*P* < 0.05, ^#^*P* < 0.001 vs. TAC.
