# Supplementary material for: Histone Deacetylase Inhibitor Phenylbutyrate Exaggerates Heart Failure in Pressure Overloaded Mice independently of HDAC inhibition
Source: Sci Rep. 2016 Sep 26;6:34036. doi: 10.1038/srep34036 (PMC5036044; doi:10.1038/srep34036)
Supplement: Supplementary Information [file srep34036-s1.pdf]

Supplementary Material

Histone Deacetylase Inhibitor Phenylbutyrate Exaggerates Heart Failure in Pressure Overloaded Mice independently of HDAC inhibition

Jing Ma, Tao Luo, Zhi Zeng, Haiying Fu, Yoshihiro Asano, Yulin Liao, Tetsuo Minamino, Masafumi Kitakaze

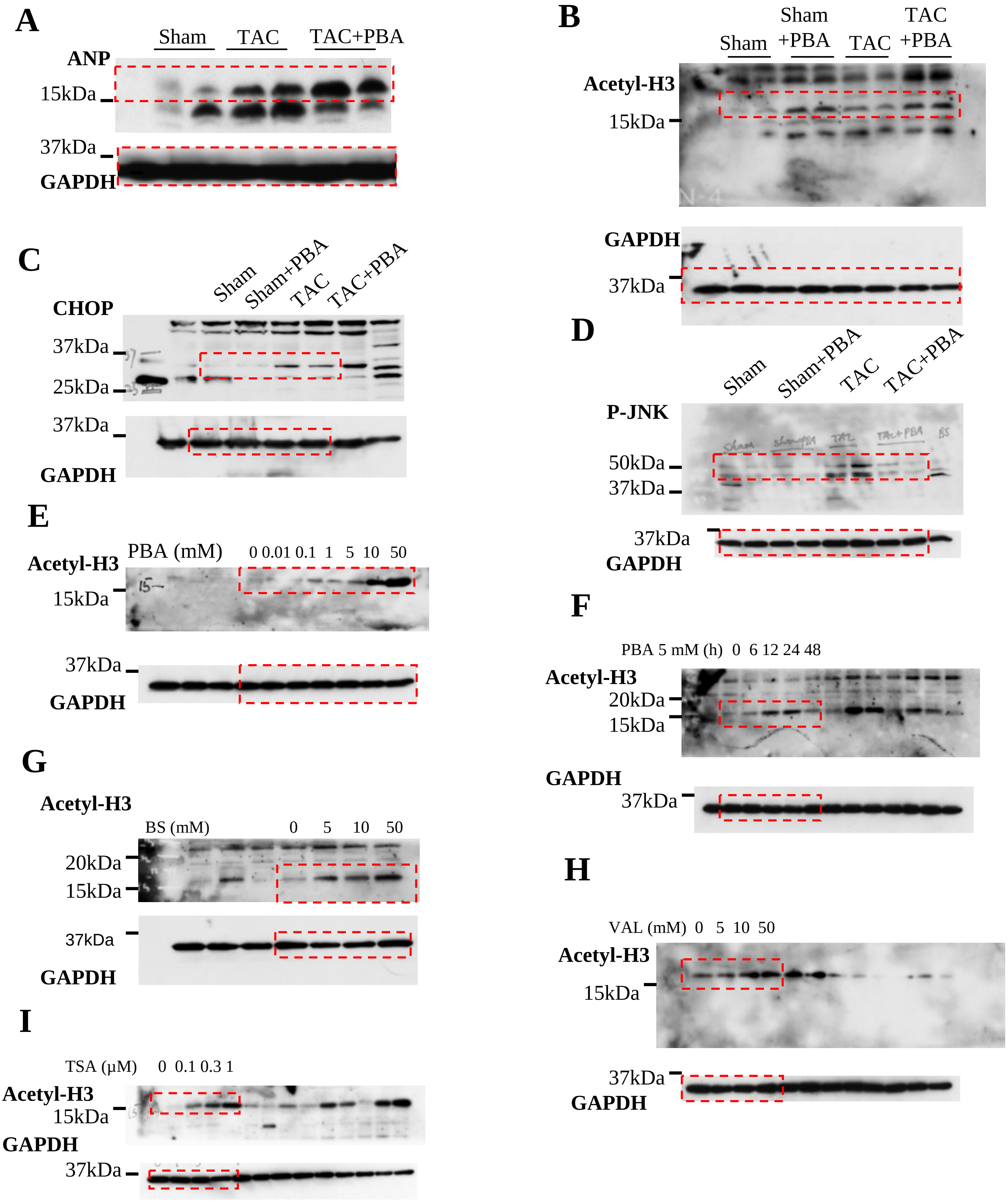

Figure S1.Original gel scan of western blot for representative cropping . A, for figure 6A. **B,** for figure 6C. **C,** for figure 6D. **D,** for figure 6E. **E,** for figure 8A. **F,** for figure 8B. **G,** for figure 8C. **H,** for figure 8D. **I,** for figure 8E.
